# Mapping Child and Adolescent Mental Health Services and the Interface During Transition to Adult Services in Six Swiss Cantons

**DOI:** 10.3389/fpsyt.2022.814147

**Published:** 2022-05-09

**Authors:** Deniz Kilicel, Franco De Crescenzo, Remy Barbe, Anne Edan, Logos Curtis, Swaran Singh, Nadia Micali, Jean-Michel Aubry, Jacqueline Mégevand, Stephan Eliez, Kerstin Jessica Plessen, Marco Armando

**Affiliations:** ^1^Department of Psychiatry, School of Medicine, University of Geneva, Geneva, Switzerland; ^2^Department of Psychiatry, University of Oxford, Oxford, United Kingdom; ^3^Child and Adolescent Psychiatry Clinical Services, Geneva University Hospital, Geneva, Switzerland; ^4^Division of Psychiatric Specialties, Department of Psychiatry, Geneva University Hospitals, Geneva, Switzerland; ^5^Warwick Medical School, University of Warwick, Coventry, United Kingdom; ^6^Office Médico-Pédagogique, Department of Public Instruction, Geneva, Switzerland; ^7^Pole Autism Foundation, Geneva, Switzerland; ^8^University Service for Child and Adolescent Psychiatry, Department of Psychiatry, Centre Hospitalier Universitaire Vaudois, University of Lausanne, Lausanne, Switzerland

**Keywords:** transition, CAMHS, AMHS, psychiatry, child and adolescent, young adults, Switzerland

## Abstract

**Rationale:**

Transition in psychiatry refers to the period where young people transit from Child and Adolescent Mental Health Services (CAMHS) to Adult Mental Health Services (AMHS). Discontinuity of care during this period is well-documented but little is known about provisions and transition characteristics and policies across Switzerland. The aim of this article is to describe the architecture of public mental health providers in Switzerland and compare it to EU countries.

**Method:**

Two mapping surveys, developed previously for European countries, were adapted and sent to cantonal experts: the adapted European CAMHS Mapping Questionnaire (ECM-Q) assessing the architecture and functioning of CAMHS and the adapted Standardized Assessment Tool for Mental Health Transition (SATMeHT) to map CAMHS-AMHS interface.

**Results:**

Data were gathered from six cantons. Activity data and transition policies were comparable between Swiss regions and European countries. The percentage of young people below 19 years who were in care was above 2% in every responding canton with a higher proportion of boys than girls for patients <12 years of age. The transition occurred at the age of 18 years, civil majority, in each canton, and between 0 and 24% (3/7) and 25% and 49% (4/7) of young people were expected to transition. One canton (1/7) benefitted from written guidelines, at the CAMHS level only, regarding transition but none had guidelines for mapping CAMHS/AMHS interface even at the regional level.

**Conclusion:**

Despite the availability of resources and even if the possibilities of access to care are on average higher than in many European countries, issues regarding transition remain comparable in six Swiss cantons when compared to Europe. Meaning that beyond resources, it is the coordination between services that needs to be worked on. Importantly, implementing those changes would not require investing financial resources but rather working on the coordination between existing teams.

## Introduction

For the last two decades, health care professionals have raised awareness on the key importance of a smooth handover for young people transiting from child to adult services when in need of a continued provision of care (1–5). Indeed, many chronic health conditions see their onset during childhood or adolescence and continue throughout the lifespan (6, 7). Nevertheless, according to the aforementioned studies, in most cases, the health care provided changes drastically between child–adolescent services and adult ones. This is also the case for many psychiatric disorders (8). These changes include a new environment, people, staff, care system, and procedures (9, 10). Indeed, Child and Adolescent Mental Health Services (CAMHS) tend to focus on developmental disorders, whereas Adult Mental Health Services treat mostly full-blown and chronic psychiatric disorders (11, 12).

The complexity and difficulty of this transition were well-documented by several studies (13). According to a recent European study including 28 countries (8), although the transition was estimated necessary for 25–49% of patients in charge at CAMHS, only 20–30% of AMHS service users under 30 had previous contact with CAMHS, underlining discrepancies in the continuity of care.

To improve this process, Forbes et al. (14) highlighted four main angles which when met lead to better continuity of care: (1) preparing the young patient for the transition, also called therapeutic intent; (2) providing a period of joint care between CAMHS and AMHS therapists, a handover period; (3) organizing transition planning meetings involving the young person, parents and/or carers, and all the professionals active in the patient's care; (4) transferring all notes and information regarding the patient including summaries from CAMHS to AMHS.

Although the above-mentioned optimal transition factors were identified 20 years ago, <5% of today's transitions meet those standards (15), leading to discontinuity of care for patients around or after transition (16).

To the best of our knowledge, Switzerland was not included in any of the studies on transition, and, more interestingly, no data on the topic are available. Nevertheless, those alarming numbers are likely also relevant for Switzerland as it displays the same rates of mental health disorders when compared to other countries for which we have data from the literature (17). Only one study requested by the Federal Office of Public Health evaluated transition strategies in Switzerland, focusing on inpatient units (18). Therefore, there is no information on transition for outpatient units which relates to most cases (19).

When considering outpatient units more specifically, it is important to understand the Swiss system and its functioning which differs from other European countries. There is no global social security and all insurances are private with basic mandatory insurance (LAMaL), usually covering all health-related costs provided by the public health domain.

In general, the medical care system grants high levels of resources and education for specialists, but mental health care for children and adolescents is the most lacking and in need of improvement. Experts even estimate that in the upcoming 5 years, Switzerland will be lacking specialized child and adolescent psychiatrists and psychotherapists (19). These numbers are even more concerning as the number of outpatient public health consultations increased by 43% between 2012 and 2018 (20). Each canton is also entitled to develop its mental health care programs with different financial, space, or staff limitations. To date, only three cantons in the German-speaking part collaborate in the development of joint care where a discussion forum was implemented aiming at information transmission and network promotion. All other cantons function on a personalized mental health care system (20).

Despite these differences between cantons, one common concern for all mental health services related to the management of young people who were reaching the transition boundary is as follows: A better understanding of how those services work and collaborate during the transition period in each canton at the outpatient unit level could benefit patients' care and might lead to the implementation of national clinical good practices. In the attempt to fill this gap of information regarding the transition from CAMHS to AMHS in Switzerland and taking into account the specificity of its aforementioned geopolitical structure, this study aims to (1) describe the architecture of CAMHS in Switzerland and (2) map the CAMHS/AMHS interface. Results will then be compared to provisions and practices in European countries other than Switzerland as transposition and replication of the studies carried out by Signorini et al. (8, 21) within the scope of the MILESTONE project. This study is also part of a larger clinical research project, the SORT study, that aims at improving the transition period in Geneva.

## Methods

### Terms

Three terms will be used extensively in this article and their definition is of utmost importance. As explained earlier, within Switzerland's psychiatric architecture, there are many private practices. For this study, all services participating are state-based and not private.

***CAMHS*** refers to Child and Adolescent Mental Health care Services that provide interventions aimed at any mental health disorder as classified by the international manuals (ICD or DSM). Those services provide any type of care from medicinal to psychosocial to individual or systemic psychotherapy and some also offer inpatient care.

***AMHS*** refers to Adult Mental Health care Services with interventions aimed at any mental health disorder as classified by international manuals (ICD or DSM). Those services provide any type of care from medicinal to psychosocial to individual or systemic psychotherapy and some also offer inpatient care.

***Transition*** refers to the moment a young person's care moves on from CAMHS to another care system with a purposeful plan taking into account multiple aspects of the young person's life (medical, social, and vocational) (15). Officially, this happens around the age of 18 years when young people are considered adults and a transfer between services must take place. Multiple outcomes exist, and this study aims at mapping where the young adults reaching the transition boundary went (i.e., end of care, private practice, and AMHS) and how well it occurred.

### Survey Participants

CAMHS directors from Swiss cantons were contacted *via* two strategies in parallel: (1) direct contact by study directors and (2) by the Swiss Society for Child and Adolescent Psychiatry and Psychotherapy (SGKJPP) (i.e., the national society for child and adolescent psychiatrists practicing in Switzerland).

In the latter, questionnaires were disseminated *via* email to each of the 26 cantons' representative chief child and adolescent psychiatrists after a presentation during a plenary meeting at the SGKJPP. The society reached out and followed up on the questionnaires' filling. The research assistant followed up with the SGKJPP and reminders (one every 2 months) were sent by the society to potential responders and another presentation was held during the society's plenary meeting to gather the missing questionnaires.

Responders were encouraged to contact the research team of the SORT study at any moment for questions or help in the process.

### Assessment Instruments

#### ECM-Q

An adapted version of the European CAMHS Mapping Questionnaire (ECM-Q; see Supplementary Material) (21) was used. Initially, the questionnaire was designed to describe and classify mental health services and measure service use (22). The ECM-Q, modified for the European MILESTONE study, integrates multiple domains including policy and legislation, health financing, CAMHS in numbers, CAMHS human resources, collaborations with other services, activity data, epidemiology and quality assurance, care for special populations, and medication modalities. Overall, the ECM-Q aims at understanding how CAMHS function from multiple points of view.

#### SATMeHT

The Standardized Assessment Tool for Mental Health Transition (SATMeHT; see Supplementary Material) (8) focuses on transition and transition-related policies. We used an adapted version to take into account specificities in Switzerland. The SATMeHT focuses on multiple aspects of transition ranging from concerning numbers, the collaboration between CAMHS and AMHS, the existence of guidelines on transition, and support systems in the process.

### Data Collection and Analyses

Each responder received both questionnaires to fill in *via* email in a Microsoft Word format (Microsoft Corporation, Redmond, WA, USA) and was asked to send them back *via* email. Missing responses were identified by DK who contacted responders to obtain missing data. Information for population by canton was extracted by DK from the governmental website of the Federal Office of Public Health (23).

Data were merged on Microsoft Excel 2016 (Microsoft Corporation, Redmond, WA, USA) and exported into SPSS (IBM SPSS Statistics for Windows, Version 27.0. Armonk, NY: IBM Corp) for cleaning and data analyses using descriptive univariate statistics (ratios, means, and standard deviations).

## Results

### Data Collection

Data were collected between November 2019 and June 2021. The SGKJPP handled questionnaire dissemination and a total of 26 child and adolescent services were approached. We received replies from six of them (response rate: 23.1%). Those six cantons represented 27.3% of the general population in Switzerland and 28.6% of young people below 19 years in 2019.

In the canton of Geneva, the CAMHS architecture presents some specificities. Indeed, there are two separate services collaborating. First, the Geneva University Hospital (referred to as HUG, Hôpitaux Universitaires de Genève), within the hospital treating specific situations (suicide attempts and ER patients) and hospitalizations and, second, the Medical-Pedagogical Office (referred to as OMP, Office Médico-Pédagogique), which is part of the department of public education and is the direct respondents to schools.

Responders filled the questionnaires based on different sources according to the type of questions asked: all accessed data logs with the help of the department's statistician and all consulted official reports or answered based on self-experience. Four out of six provided references for their sources (e.g., internal reports, websites, and cantonal publications).

### CAMHS Provision and Functioning

The number of outpatient units for each canton ranged between one and four. On average, there were 3.13 units per 100,000 people below 19 years with the lowest rate at 1.13 and the highest at 5.91 (see Table 1). CAMHS was accessible between 8 and 10 h per working day (mean = 8.58) and two cantons out of six offered an emergency CAMHS team working outside CAMHS service hours, with one being mobile.

**Table 1:** Demographics and child and adolescent service provision for cantons having filled the questionnaires (*N* = 7).

	**Total population^**a**^**	**Proportion of population below 19^**a**^**	**Number of public CAMHS^**b**^**	**Number of inpatient psychiatric beds^**b**^**	**Number of child and adolescent psychiatrists^**b**^**	**Number of child and adolescent psychologists^**b**^**
Basel-City	195 844	17.30%	2.95	118.06	NA	NA
Geneva	504 128	21.00%	1.89	26.45	66.12	125.63
HUG	-	-	-	26.45	35.89	29.28
OMP	-	-	-	0	30.23	96.35
Fribourg	321 783	22.20%	4.20	12.60	14.00	NA
Neuchâtel	176 496	21.00%	2.70	16.19	40.47	80.94
Valais	345 525	19.60%	5.91	17.72	4.43	5.91
Vaud	805 098	21.90%	1.13	13.61	NA	NA

*CAMHS, child and adolescent mental health services; HUG, Hôpitaux Universitaires de Geneve; OMP, Office Medico-Pedagogique; ^a^Data from the Federal Office of Statistics; ^b^for 100,000 people below 19 years of age; NA, information was not available; -, information in global canton*.

Each CAMHS also offered inpatient units. Those ranged between 12.60 and 118.06 beds with a mean value of 34.10 beds for 100,000 young people (0–18 years old; see Table 1). Each CAMHS also could use multiple pediatric beds for psychiatric patients if needed. The number of child and adolescent psychiatrists in each CAMHS varied from 4.43 to 40.47 with a mean of 25.00 psychiatrists for 100,000 young people. Regarding the number of child and adolescent psychologists in public CAMHS, there was a mean of 53.12 psychologists for 100,000 young people below 19 years, from 5.91 to 96.35 (Table 1).

All responding cantons benefitted from a juvenile justice system, where five out of seven also offered access to a specialist in mental health. All cantons provided educational services for children and adolescents with special needs and four reported it as excellent. Two cantons out of six judged community-based outpatient care as available and excellent for most situations, whereas the other three rated them as either absent, insufficient, or of low quality. Three out of six offered day patient programs where only one judged it as excellent. Group homes and respite care placement were available in one canton. All cantons offered language interpreters for diagnostic assessment and care available in all geographic areas for half the cantons and fewer in the other half.

Specific subgroups of children and adolescents also had access to specifically tailored mental health services. Three out of seven have programs designed for children with autism spectrum disorders or the seriously emotionally disturbed; three for refugees, two for children affected by natural or man-made disasters, and one for runaway or homeless children. One canton had no specifically designed mental health service.

Considering CAMHS's collaboration with other services, most cantons reported having a protocol in place with schools to refer children with suspected learning disabilities. All cantons had a high presence of protocol for children victims of abuse and/or neglect with a satisfactory relationship between CAMHS and minor protection services. One canton reported having an official procedure for crossovers between primary and secondary care. All cantons had family/carer's associations and six out of seven also had service user's associations (for more detailed information please see Appendix 1).

All cantons displayed different financing proportions for mental health care. All benefitted, at different levels, from the participation of the Swiss Federal Law on Compulsory Health Care (LAMaL), which is the mandatory health care insurance for all people living in Switzerland. It is offered by private insurance companies and represents the minimal standard for health care insurance. Its contribution to mental care financing ranged between 25 and 80%. Most of the remaining financing was supported by tax-based cantonal government funding (from 40% up to 70%, one canton did not benefit from it; for more detailed information, please see Appendix 2).

### CAMHS Activity Data and Quality Assurance

**Figure 1:**
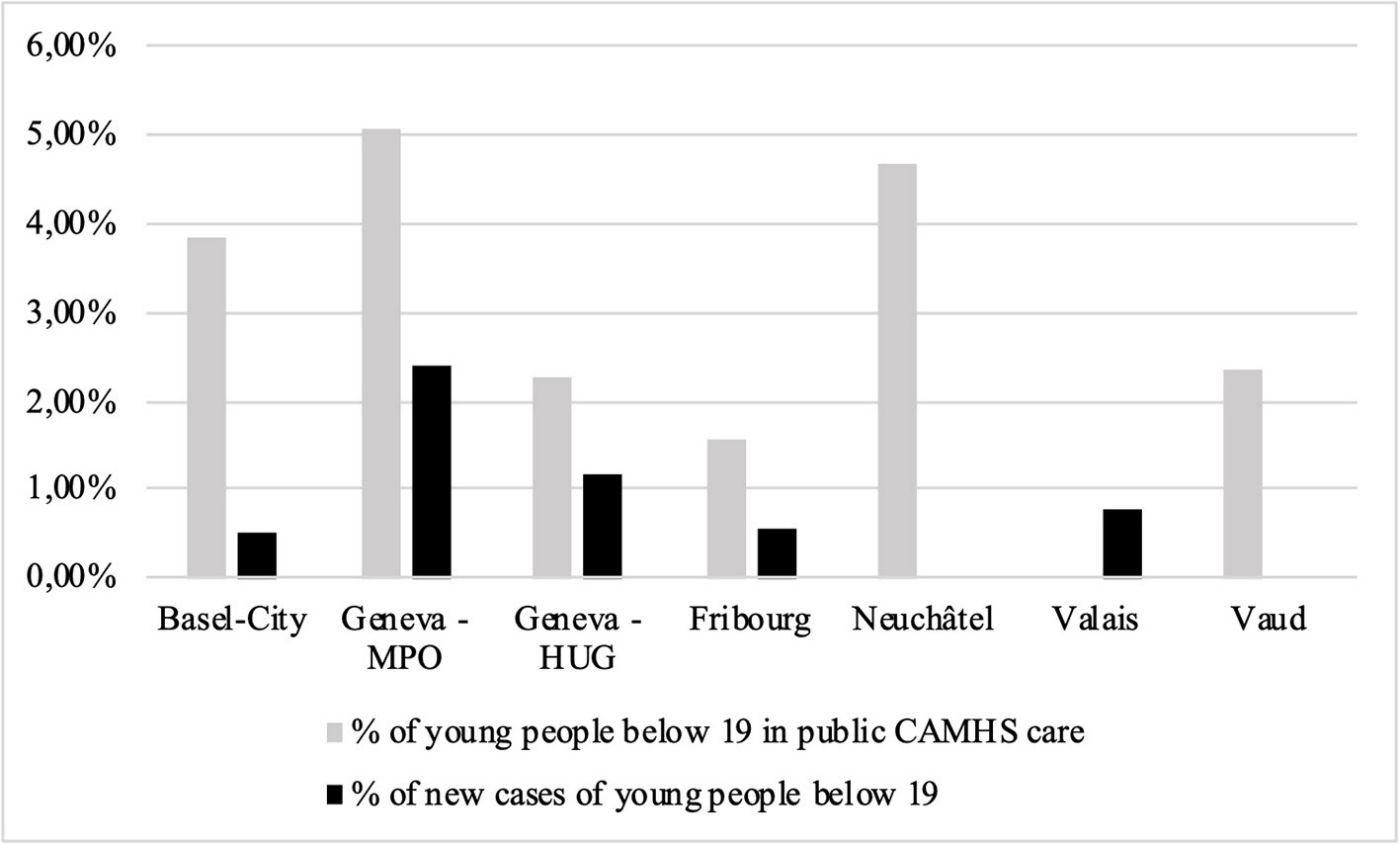
Proportion of young people below 19 years who were in CAMHS care in 2019, over the total number of people below 19 years in each canton. The percentage of new yearly cases (in black) is also included in the total. Empty columns represent missing data. CAMHS, child and adolescent mental health service; HUG, Hôpitaux Universitaires de Geneve; OMP, Office Medico-Pedagogique.

The proportion of young people treated in CAMHS ranged between 1.6 and 5.1% of the general population below 19 years of age with an overall mean of 3.2%. All cantons had higher rates of male service users than females; on average, 55.2% were males (SD = 0.03) and 44.8% females (SD = 0.03). Newly recorded cases were available for five cantons and ranged between 0.5 and 2.4% of the population below 19 years of age (Figure 1).

Four cantons provided specific numbers with detailed age ranges. It appeared that in three cantons, most patients were children under 12 years (74.0, 66.4, and 56.9%, respectively), whereas in the other, patients were mostly above 12 years (59.1%).

**Figure 2:**
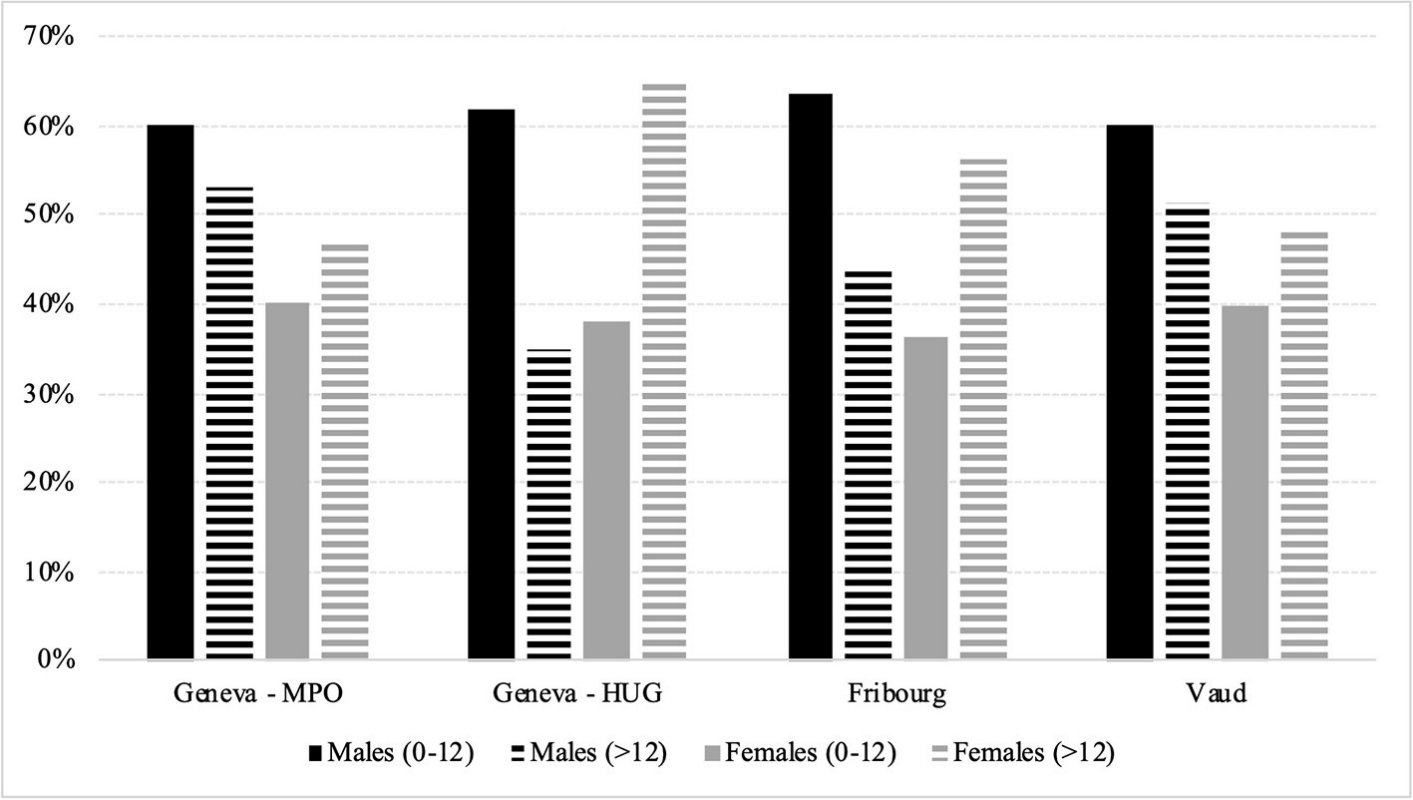
Young people below 19 years in CAMHS care in 2019 for available cantons (4/6), percentage by age range separated by gender. HUG, Hôpitaux Universitaires de Geneve; OMP, Office Medico-Pedagogique.

Although the global CAMHS trend showed higher rates for males, the need for care in males was higher for children under 12 years of age with 61.4% of boys compared to 38.6% of girls. For patients above 12 years, the percentage of males dropped down between 34.9 and 53.1% (Figure 2). Five out of seven cantons reported having a mandatory periodic activity report and two had no requirement. We did not have access to the reports. Cantons (4/7) also reported having an epidemiological data collection system for child and adolescent mental health disorders but the data were only available for one canton, partially for a few specific disorders for the second and not for the last two. Service data were collected in half the cantons as well but only for inpatient units.

Health policies, including mental health in four cantons, were available at the cantonal level and six out of seven cantons had a mental health policy covering young people up to their 18^th^ birthday. For more detailed information on the components included in the policies, please see Appendix 3.

All cantons benefitted from specific laws regarding child and adolescent protection. Most commonly used medications were consistent across cantons. Data are missing for two cantons (for more detailed information, please see Appendix 4).

All cantons reported national minimum standards for professional certification and maintenance of competencies for child and adolescent psychiatrists and psychologists with in-service training and clinical supervision. Four out of seven had clinical guidelines for both professions.

### Transition Process and Service Configuration

In three cantons, the transition was estimated to occur for 0–24% of young people treated in CAMHS, while from 25 to 49% in the other four. Those estimations were concordant with the estimated percentage of AMHS service users below 30 with previous CAHMS experience (5). In one canton, this percentage (25 to 49%) exceeded the expected need for CAMHS service users' transition (0 to 24%).

In most cantons (five out of seven), CAMHS and AMHS were organized separately. The two that had an integrated organization also applied specialized transition planning at the age of 18 or even 21 years for one canton. The transition occurred officially in all cantons at 18 years, but for one at the age of 17. The two cantons applying an official transition planning implicated all actors of the patient's care: child psychiatrist, psychologist, nurse, social worker, speech therapist, and physiotherapist. Regarding guidelines, one canton had a written policy for transition from CAMHS to AMHS, but no policy existed for managing the interface between services.

Financially, budgets to help transition support services were shared between CAMHS and AMHS in one canton, whereas the other cantons had separate funding systems.

Three out of four had CAMHS case managers who could still work with the young adults after they transitioned to adult services. Parents/carers also had an active role in transition decisions and service choice (6/7), whereas they received information about the procedures but had no involvement in one. If the patient's parent was considered to have mental health issues leading to poor judgment, the CAMHS team could consult other individuals or instances regarding transition decisions, more specifically child protection services and family doctors (5/6), the parent's psychiatrist (3/6), the other parent or parent's partner (3/6), and the AMHS psychiatrist (2/6). The parent with a mental disorder was informed about transition procedures in four cantons and could play an active role in two. When a physical disease was coexisting, four out of six cantons managed transition by referring or in consultation with the appropriate specialist of the comorbidity. In two cantons, the CAMHS clinician stayed in charge of transition decisions and young people were expected to stay longer in pre-transition care. One canton also reported, to the best of their knowledge, never having encountered this situation.

Overall, no canton had a standardized assessment of the needs for transition services but four out of seven underlined efforts from AMHS teams to address young people's needs with growing CAMHS/AMHS collaborations, internal procedures, and projects.

### Transition Programs and Actions

Programs dedicated to transition varied significantly among cantons. All cantons reported the lack of a structured transition in support of the transition process to AMHS and of support in the patient's adult life. However, several aspects of transition were planned: a joint meeting between CAMHS and AMHS teams, involvement of the parent/carer in the care plan, involvement of the young person in the decision-making, preparation of the young person for the end of the therapeutic relationship, and having one accountable clinician for the transition process (see Appendix 5). No canton had a transition team dedicated to this period. Three out of seven cantons had joint work between AMHS and CAMHS service providers and adult service providers in many areas. Two cantons involved the young person active in the planning, while three others also used shared documentation and record-keeping systems (Appendix 6).

Cantons also provided additional programs to address young people's needs during the transition. These included supervised or supported housing (3/7), vocational support and supported education (2/7), standard global support, assertive community treatment, and health promotion (1/7). Globally missing possible supports were specialized wraparound approaches, independent living preparation, mentoring, and a transition specialist (see Appendix 7).

## Discussion

This article, through surveys adapted from two European studies (8, 21), aimed to better describe the architecture of CAMHS in Switzerland and map the CAMHS/AMHS interface around the transition period in light of the care provided in European countries (8).

Results showed a wide range of financing proportions and service distribution between cantons in terms of available beds and professionals as well as the number of facilities and user access to mental health care. Although most cantons had a mandatory periodic activity report, only one could provide specific diagnostic information, which was not shown here. Specific care systems were mostly available (i.e., the juvenile justice system, educational services, day patient programs) and collaborations with schools acting as referrals benefitted from protocols in almost all participating cantons. Legal aspects such as child protection and minimum training standards for clinicians were homogenous as they are ruled by federal authorities, at the country level.

Considering the heterogeneity of answers provided regarding the type of care, policies, programs, human resources, and tools used in the cantons, we can conclude that the management of mental health care between different cantons in Switzerland resembles the architecture in Europe, although geographically smaller (8). Throughout the following section, answers provided in Switzerland will be compared to the results of the 28-country European surveys (see Table 2 for a summary).

**Table 2:** Comparison between regional Swiss canton and European countries.

Provision and functioning	N° of units	CH = EU
	N° of beds	**CH > EU**
	N° of psychiatrists	**CH > EU**
	N° of psychologists	**CH > EU**
Activity data	Adolescents in care	CH = EU
	Gender distribution	CH = EU
	New cases per year	CH = EU
	Specific care (autism and emotional disturbances)	CH = EU
	Not optimal care (trauma, refugee, runaway, homeless)	CH = EU
Transition	Lack of guidelines	CH = EU
	Lack of specifically tailored transition	CH = EU
	Lack of CAMHS/AMHS communication	CH = EU

*AMHS, adult mental health services; CH, Switzerland; CAMHS, child and adolescent mental health services; EU, European Union (data from the MILESTONE project)*.

### Child and Adolescent Mental Health Services

Swiss CAMHS provisions were as varied as in separate European countries in terms of the number of CAMHS and beds available for child and adolescent psychiatric service users. In Switzerland, the canton showing the highest number of CAMHS for 100,000 young people below 19 years was also the one with the widest catchment area and hard-to-reach regions. Psychiatric inpatient beds for 100,000 young people seemed to be generally higher in Switzerland compared to European countries, although the span was very broad in participating cantons. Specific statistics could not be retrieved, but when looking at inpatient psychiatric beds globally, we could define that cantons in the German part have more beds available compared to the French or Italian region. In line with our results for pediatric psychiatric beds, Basel-City offered more beds compared to all other participating cantons, with Fribourg having the lowest (217 beds available for 100,000 people from the whole population, compared to 53) (23).

One of the great differences was the number of psychiatrists available for 100,000 young people below 19 years of age. Whereas, in Europe, more than half of the countries had <10 (16/27) and four out of five cantons had 10 and more. This could be explained by the education system in Switzerland, where child and adolescent psychiatrists must complete their training under the supervision of senior clinicians within cantonal public services before they complete their diplomas. Despite a higher presence of the child and adolescent psychiatrists, there is still a need for more professionals in Switzerland with, currently, long waiting lists and sometimes adult psychiatrists having to take on adolescent patients (18). Psychologists were also mostly more present in Switzerland compared to European countries.

The proportion of young people below 19 years of age in CAMHS care for each canton was comparable to results found in 8/19 European countries (proportions above 2%). As previous literature shows (WHO, 2002, Gender and mental health), before the age of 12, the proportion of male patients was higher than females (conduct disorders and antisocial behaviors). After 12 years, this trend is either more balanced or even flips with sometimes more girls than boys (depression and eating disorders). The most striking difference was displayed by the HUG with great differences when comparing patients' gender before and after 12 (62% of males before 12 vs. 35% after 12). This could be explained by the global architecture of CAMHS in Geneva as well. More specifically, the HUG is the service offering most of the care for children below 4 years with a specific service within the hospital, and also offers a unique institution to prevent suicides in young people. Those two approaches do not exist at the OMP and children are referred to the HUG. As the youngest patients are more often males and older one's females, this could explain the discrepancy we observed.

As in Europe, the availability of CAMHS and clinicians were not in compliance with the prevalence of patients in care which seemed to depend more on external factors (i.e., financial and care culture) rather than the population's needs.

In participating cantons, the collaboration of CAMHS with other services (juvenile justice system, patients with special needs, schools, minor protection services, interpreters, and associations) was mostly present and satisfactory. Those collaborations occurred in most cantons without a protocol in place relying on professionals' will, knowledge, and time when needed. Disorders with the most present specific care system for children and adolescents were autism spectrum disorders or serious emotional disturbances. As for the European countries, the needs of children and adolescents undergoing special circumstances (affected by natural or man-made disasters, refugees, and runaway or homeless) were not optimally met.

In summary, provisions and care needed in the participating cantons in Switzerland appeared comparable to European countries. However, young service users in Switzerland seemed to have easier access to mental health professionals showing higher numbers of psychiatrists, which can be imputed to the health financing and education specificities in the country.

### Transition From CAMHS to AMHS

In line with previous findings on inpatient transitional care in Switzerland (18), there were no official guidelines or policies in place for the transition period in outpatient units, although a substantial number of service users in CAMHS care were expected to be concerned. Some initiatives do exist within cantons and between services but stay at a local level and depend on the health care professionals' knowledge of the system. Moreover, CAMHS and AMHS mostly lack connections with different services, funding, and care systems leading young patients to experience a whole new environment and care, although those were crucial aspects highlighted for an improved transition experience (14).

According to the surveyed cantons, there is a lack of support for the management of the transition period, and the young person and family/carer are not much involved in the process, even though engaging service users in their care path have shown improvement in their understanding and acceptance of care (14).

Taken together, our results show the need for improvement in transitional care not only in inpatient units (18) but also in outpatient ones. Typically, and as for European countries are concerned, the two most crucial aspects of transition management were not met as there was no individually tailored transition program nor was there connection and homogeneity between CAMHS and AMHS.

More generally, we have tried to summarize the key ingredients of an effective transition in Table 3. There are multiple aspects to consider when approaching a transition period. Possible factors of a suboptimal transition include the lack of a clear plan and specific transition units, as well as factors related to the diagnostic (24). Individual factors inherent to the patient also impact the quality of the transition, which include: lack of perceived control over the situation, will of the patient to take part in the process, and making the transition decision based on chronological age rather than developmental age (25). On the other hand, multiple elements are essential for a smooth transition: coordination and early planning between CAMHS and AMHS and involvement of the patient and caregivers in the transition process and decision (26). Other factors that have proven efficient are the presence of a reference adult or case manager (pre- and post-transition) as well as the continuity and consistency of care.

**Table 3:** Summary of key elements to take into account for a smooth transition.

Essential elements for an optimal transition	Possible causes of a suboptimal transition
Early planning	Lack of a clear plan
Coordination between pre- and post-transition professionals	Impact of the diagnosis
Involvement of the young patient and the family or carers in the decisions	Lack of transitional psychiatry units or of an appropriate destination
Continuity and consistency of care	Factors inherent to the individual ✓ Failure to take into account developmental age rather than the chronological one ✓ Lack of control over the situation for the patient ✓ Lack of willingness to take part in the decision by the patient
Presence of a case manager or reference professional	

### Limitations

One major limitation of our study is that despite the strategy that has been developed to reduce any reporting bias, only six cantons (out of 26) participated in the study with a majority of French-speaking cantons leading the results to be taken cautiously in terms of validity and generalizability. As mentioned previously, Switzerland is a geographically and population-wise small country with mainly three different spoken languages (German, French, and Italian). Those languages also generally lead to artificial boundaries where information sharing and transfer are difficult. Moreover, as underlined by previous federal reports, in Switzerland, precise information on mental health numbers and policies is lacking (18, 19). However, general reports from the Federal Office of Statistics (23) seem to point out some similarities between German- and French-speaking regions. As an example, the percentage of money allocated to psychiatric care ranges from 8 to 12% with an overall mean of 10% for Switzerland and a different proportion for the Italian-speaking part with 5% of health-related expenses directed to psychiatry. This indicates that although few cantons replied, results might still reveal important knowledge. Other reasons could also explain the lack of response from other cantons, those being cultural difficulties to report on a delicate issue or the length of the survey and the type of questions.

The questionnaires also referred to sensitive topics where answers' variability might be caused by responders' attitudes or misunderstanding of some definitions that were provided with the survey. Although they were free to contact the research team anytime, only one requested a meeting to clarify some aspects.

Moreover, many of the specific questions included missing data which was not systematically recorded by the services. Those were then inferred by the closest information provided.

## Conclusion

Despite greater resources available for mental health services around Switzerland as compared to Europe, our findings show that Swiss cantons experience similar problems and needs when considering transition policies. A possible interpretation is that beyond the resources invested in mental health care, coordination between services is the most important aspect to be addressed. It seems therefore crucial to create nationwide guidelines to better support transition periods for young people which would then be adapted to the cantonal specificities. These coordination policies would also not need any major financial burden on the mental health system as they do not require more workforce.

## Data Availability Statement

The raw data supporting the conclusions of this article will be made available by the authors, without undue reservation.

## Author Contributions

DK, SE, KP, and MA disseminated the questionnaires. DK collected the data and analyzed it with FD. RB, AE, LC, NM, J-MA, JM, and KP offered support for clinical aspects of the manuscript with their combined expertise. DK, FD, LC, and MA wrote the manuscript. All authors proofread and approved the manuscript.

## Funding

FD was supported by the National Institute for Health Research (NIHR) Research Professorship to Professor Andrea Cipriani (grant RP-2017-08-ST2-006) and by the NIHR Oxford Health Biomedical Research Centre (grant BRC-1215-20005). Open access funding was provided by the University of Lausanne.

## Author Disclaimer

The views expressed are those of the authors and not necessarily those of the UK National Health Service, the NIHR, or the UK Department of Health.

## Conflict of Interest

FD is an employee of Boehringer Ingelheim International GmbH (from February 2022). DK is an employee of Syneos Health GmbH (from March 2022). The remaining authors declare that the research was conducted in the absence of any commercial or financial relationships that could be construed as a potential conflict of interest. The Handling Editor AR declared a past co-authorship/collaboration with one of the authors MA.

## Publisher's Note

All claims expressed in this article are solely those of the authors and do not necessarily represent those of their affiliated organizations, or those of the publisher, the editors and the reviewers. Any product that may be evaluated in this article, or claim that may be made by its manufacturer, is not guaranteed or endorsed by the publisher.
