# Erratum: Mapping child and adolescent mental health services and the interface during transition to adult services in six Swiss cantons

**DOI:** 10.3389/fpsyt.2023.1186126

**Published:** 2023-03-24

**Authors:** Frontiers Production Office

**Affiliations:** Frontiers Media SA, Lausanne, Switzerland

**Keywords:** transition, CAMHS, AMHS, psychiatry, child and adolescent, young adults, Switzerland

An omission to the funding section of the original article was made in error. The following sentence has been added: “Open access funding was provided by the University of Lausanne.”

The original article has been updated.

