# Supplementary material for: Mapping Child and Adolescent Mental Health Services and the Interface During Transition to Adult Services in Six Swiss Cantons
Source: Front Psychiatry. 2022 May 9;13:814147. doi: 10.3389/fpsyt.2022.814147 (PMC9125157; doi:10.3389/fpsyt.2022.814147)
Supplement: Supplementary file 1 [file Data_Sheet_1.PDF]

# SORT STUDY

# Standardized Assessment Tool for Mental Health Transition

# SATMeHT

## Location Information

Name of Canton:

Date of Form Completion: Month________Year__________

## Contact Details of Person Responsible for Answering Questionnaire

Name:

Title:

Position:

Mailing Address:

Telephone:

E-mail:

## Please provide an estimate if official data is unavailable.

Canton population:

Canton population under the age of 18:

## Instructions:

This questionnaire has been developed to provide information about the current state of organization of Youth Mental Health Care Services across all Switzerland, especially for the delicate process of transition from Child and Adolescent Mental Health Services and Adult Services. As part of the SORT project the aim of this instrument is to collect country specific information on transitional care and map strengths and weaknesses of it at a Swiss level.
Please provide responsibly an answer for all the items referring to what typically happens in your canton and feel free to consult with other experts, where necessary. Many questions have a choice of multiple answers: please circle appropriate responses or write in the space provided, where required. You can also write specific comments in the section NOTES in the case you feel more details need to be provided.
Once the questionnaire is completed you can submit it by post or email to study the coordinator.
In case you need any further clarification please do not hesitate to contact the research team.

We also thank the MILESTONE Consortium for developing this questionnaire.

**Legend:**

- CAMHS= Child and Adolescent Mental Health Services (specialist, community-based, multidisciplinary, mental health services delivering medical and psycho-social interventions for children and adolescents with mental health problems and disorders and/or neuropsychiatric/developmental disorders
- AMHS=Adult Mental Health Services
- By transition/transitional care we mean health care transition defined as a formal transfer of care from CAMHS to adult services.

1. Roughly what percentage of CAMHS service users in your canton need specialist adult mental health services as they get older?
   a. 0-24%
   b. 25-49%
   c. 50-74%
   d. 75-100%

1.1. On which basis have you formulated this estimate?
   a. On a published study on this topic carried out in your canton/country
   b. On national/regional register data
   c. On local register data
   d. On personal opinion/experience
   e. Others (please specify) ______________________

2. What is the likely percentage of patients under 30 years of age who access AMHS with prior contact with CAMHS in their history? ________%

3. Are CAMHS and AMHS organized separately or are they directed by one unique manager?
   a. Separate services
   b. Integrated services

4. Are there any regional differences in the configuration of CAMHS and AMHS?
   a. Yes
   b. No

5. What is the transition age in healthcare including mental health care? *(Please indicate two ages if the two sectors are differing)* ___________________

6. If specialized transition planning in mental health care happens, at what age(s) in year does it most often occur? ________/ ☐ There is no specialized transition planning

   6.1. Who is involved? *(Please circle all that apply)*
   a. Child psychiatrist
   b. Psychologist
   c. Nurse
   d. Social worker
   e. Logopedics
   f. Physiotherapist
   g. Other (please specify): ____________________________

7. Do you have a written policy or guidelines at a national or regional level for transition of patients under CAMHS care to adult services?
   a. Yes (*please attach a copy or provide link/reference to the document*)
   b. No

8. Do you have a written policy or guidelines at a national or regional level for managing the interface (i.e. the point at which interaction occurs) between CAMHS and adult services?
   a. Yes (please attach a copy or provide link/reference to the document)
   b. No

*9.* What are the budgetary and fiscal factors that aid transition support services and efforts? *(Please check all that apply)*
   a. Availability of flexible funding
   b. Financial agreement with private services
   c. Different funding levels
   d. Separate funding of Child/Adult Mental Health Services
   e. Other *(please specify)* ______________________

10. Can CAMHS case managers still work with young adults that have begun receiving adult mental health care?
    a. Yes
    b. No
    c. Case manager not available – *a case manager is for example a nurse or educator who knows the young adult very well from the CAHMS service*

11. What is the role of parents for users who face the transition from CAMHS to AMHS?
    a. They are informed about the procedures and the characteristics of the transition process
    b. They play an active role in choosing the service of referral
    c. They play an active role in choosing the treating clinician at the service of referral
    d. No/limited involvement *(if requested by the patient)*
    e. Other *(please specify)* ____________________

*12.* If in the service there are any concerns about a CAMHS user's parent with a mental health problem, which professionals or agencies would most likely be involved?
*You may wish to consult with the list below*
    a. ____________________________________________
    b. ____________________________________________
    c. ____________________________________________

| | |
|---|---|
| Clinical supervisor | Parent's adult key worker |
| Mental Health Social Worker | Child & Family Social Worker |
| Family Doctor (GP) | Child Protection Team |
| Parent's partner/spouse | Parent's Psychiatrist |
| Parent's Community Psychiatric Nurse | Other (please specify) |

13. In the case of a parent with a mental health problem what is his/her role in the transition process?
    a. He/she's informed about the procedures and the characteristics of the transition process
    b. He/she plays an active role in choosing the service of referral
    c. He/she plays an active role in choosing the treating clinician at the service of referral
    d. He/she is left alone to manage it by him/herself

*14.* What sorts of difficulties are children or young people, who need transitional care and have mental disorders, most frequently personally experience? *(Please check all that apply)*
    a. Lack of connection between CAMHS and AMHS
    b. Lack of specific competencies in AMHS
    c. Lack of specific destination
    d. Full AMHS caseload
    e. Eligibility differences
    f. System culture differences
    g. Ignorance of other systems
    h. Territoriality
    i. Other *(please specify)* __________

*15.* How is transition managed when a physical disease/disorder is co-existing? *(Please check all that apply)*
    a. Choice in mental health specialist
    b. Communication/transfer to the colleague
    c. Keeping the medical treatment in own hands for longer
    d. Transferring to a colleague in adult care sooner
    e. Other *(please specify)* ____________

16. Has a standardized assessment of needs been done for transition services aimed at the young adult population?

    a. Yes
    b. No

        16.1. If yes, state-wide or locally?

            a. State-wide
            b. Locally

17. Have AMHS made any efforts to address the transition needs of CAMHS service users?

    a. Yes (*please specify*________________________________________)
    b. No

18. Does your canton employ any of the following to support the child during transition to adult services? *(Please tick all categories that apply: 1. No areas, 2. Few areas, 3. Many areas, 4. Most areas, 5. All areas)*

| | | | | | | |
|---|---|---|---|---|---|---|
| 18.1 | A transition team (a half-way house to support young people to access adult services) | 1 | 2 | 3 | 4 | 5 |
| 18.2 | Joint working with adult service providers | 1 | 2 | 3 | 4 | 5 |
| 18.3 | Joint working between adult and children's service providers | 1 | 2 | 3 | 4 | 5 |
| 18.4 | Partnership approaches involving the young person in planning their own transfer to adult services | 1 | 2 | 3 | 4 | 5 |
| 18.5 | Shared documentation and record keeping system | 1 | 2 | 3 | 4 | 5 |
| 18.6 | Use of out-of-clinic support groups to support young people as they move into the less structured life of adulthood | 1 | 2 | 3 | 4 | 5 |
| 18.7 | Other (please specify):___________ | 1 | 2 | 3 | 4 | 5 |

19. Are the service listed above, available in medical care and/or mental health care? *(Please tick all categories that apply)*

| | | **Medical** health care | **Mental** health care |
|---|---|---|---|
| 19.1 | A transition team (a half-way house to support young people to access adult services) | | |
| 19.2 | Joint working with adult service providers | | |
| 19.3 | Joint working between adult and children's service providers | | |
| 19.4 | Partnership approaches involving the young person in planning their own transfer to adult services | | |
| 19.5 | Shared documentation and record keeping system | | |
| 19.6 | Use of out-of-clinic support groups to support young people as they move into the less structured life of adulthood | | |
| 19.7 | Other (please specify):___________ | | |

20. Do mental health services offer programs in the following areas that address the needs of youth or the young adult population? *(Check programs that are funded and provided in at least 50% of CAMHS in your canton)*
    a. Supervised or supported housing
    b. Specialized wraparound approaches (tailored to address transition needs)
    c. Standard wraparound approaches
    d. Vocational support
    e. Independent living preparation
    f. Supported education
    g. Peer leadership/mentoring
    h. Transition specialist
    i. Assertive community treatment
    j. Other (please specify) ________________________
    k. No transition support services

21. If any of the programs above are offered, what evidence has been produced to highlight their effects? *Please provide references to any national/international publications.*

______________________________________________________________________

______________________________________________________________________

______________________________________________________________________

______________________________________________________________________

22. For patients making a transition, does your policy mainly aim for... *(please indicate frequencies of the objectives from the list below: 1. Never, 2. Sometimes, 3. Always*)

| | | | | |
|---|---|---|---|---|
| 22.1 | Documented hand-over planning | 1 | 2 | 3 |
| 22.2 | Joint meeting with adult service | 1 | 2 | 3 |
| 22.3 | Involvement of parents/carers in care plan and decision making | 1 | 2 | 3 |
| 22.4 | Involvement of service users in care plan and decision making | 1 | 2 | 3 |
| 22.5 | Preparing the young person for ending one therapeutic relationship and starting | 1 | 2 | 3 |
| 22.6 | Accountability for the process (e.g. a single clinician may be identified from one of the services to co-ordinate the transition). | 1 | 2 | 3 |
| 22.7 | Other (please specify) ____________________ | 1 | 2 | 3 |
