# Supplementary material for: Mapping Child and Adolescent Mental Health Services and the Interface During Transition to Adult Services in Six Swiss Cantons
Source: Front Psychiatry. 2022 May 9;13:814147. doi: 10.3389/fpsyt.2022.814147 (PMC9125157; doi:10.3389/fpsyt.2022.814147)
Supplement: Supplementary file 2 [file Data_Sheet_2.PDF]

SORT STUDY

European CAMHS Mapping Questionnaire ECM-Q

Location Information

Name of canton :

Date of Form Completion: Month________Year__________

Contact Details of Person Responsible for Answering Questionnaire

Name:

Title:

Position:

Mailing Address:

Telephone:

E-mail:

Please provide an estimate if official data is unavailable.

Country population:

Country population under the age of 18 (or the age of majority):

Instructions:

This questionnaire has been developed to provide information about the current state of organization of Youth Mental Health Care Services across all Switzerland, especially for the delicate process of transition from Child and Adolescent Mental Health Services and Adult Services. As part of the SORT project the aim of this instrument is to collect country specific information on transitional care and map strengths and weaknesses of it at a Swiss level.
Please provide responsibly an answer for all the items referring to what typically happens in your canton and feel free to consult with other experts, where necessary. Many questions have a choice of multiple answers: please circle appropriate responses or write in the space provided, where required. You can also write specific comments in the section NOTES in the case you feel more details need to be provided.
Once the questionnaire is completed you can submit it by post or email to study the coordinator.
In case you need any further clarification please do not hesitate to contact the research team.
We also thank the MILESTONE Consortium for developing this questionnaire.

POLICY AND LEGISLATION

1.1. Does your country/canton have an official national/cantonal child and adolescent mental health policy?

a. Yes (*underline if CANTONAL or NATIONAL)*

b. No

1.1.1. If yes, what are the age-ranges included in this policy? ________________________

1.2. What are the components of this policy?

1.2.1	Regulations on type of care provided	Yes	No
1.2.2	Regulations on competency of care providers	Yes	No
1.2.3	Specific, written standards of service provision<br>(i.e., outpatient and inpatient facilities per 100,000 inhabitants, number of psychiatrists/psychologists per 100,000 inhabitants, other service provisions)	Yes	No
1.2.4	Guidelines regarding access to services	Yes	No
1.2.5	Other (please specify) _______	Yes	No

1.3. In which type of policy is it addressed? *(Please check all that apply)*

a. Mental Health

b. Health

c. Human Rights

d. Child Protection

e. Health Social Welfare

f. Other (please specify) : ___________________________

Please enclose a copy of the relevant policy section(s).

1.4 Does any law protect children & adolescents in terms of:

1.4.1	Abuse or exploitation by adults	Yes	No
1.4.2	Confidentiality of health care services and records	Yes	No
1.4.3	Informed consent (direct or via primary caregiver)	Yes	No
1.4.4	Prescription of medications or other medical treatments	Yes	No
1.4.5	Participating in experimental trials	Yes	No
1.4.6	Other (please specify) : ____________________	Yes	No

1.5 Are there national/cantonal minimal standards of care expected from psychiatrists working in child and adolescent mental health services?

a. Yes (*underline if CANTONAL or NATIONAL)*
b. No

1.5.1 How are standards maintained? *(Please check all that apply)*

a. Professional certification and maintenance of competency
b. In-service training
c. Clinical supervision
d. Clinical practice guidelines
e. Other *(please specify)*: ______________________________

1.6 Are there national/cantonal minimal standards of care expected from psychologists working in child and adolescent mental health services?

a. Yes
b. No

1.6.1 How are standards maintained? *(Please check all that apply).*

a. Professional certification and maintenance of competency
b. In-service training
c. Clinical supervision
d. Clinical practice guidelines
e. Other *(please specify)*: ______________________________

1.7 Are there national/cantonal minimal standards of care expected from nurses working in child and adolescent mental health services?

a. Yes
b. No

1.7.1 **How are standards maintained?** *(Please check all that apply).*

a. Professional certification and maintenance of competency
b. In-service training
c. Clinical supervision
d. Clinical practice guidelines
e. Other *(please specify)*: ______________________________

1.8 Are there any standardized evaluation procedures to assess the functioning of services?

1.8.1	Outcomes (individual health, individual functioning)	Yes	No
1.8.2	Patient satisfaction	Yes	No
1.8.3	Family satisfaction	Yes	No
1.8.4	Other *(please specify)*	Yes	No

---

2. HEALTH FINANCING

2.1. How are child and adolescent mental health services funded (excluding specialized services for children with mental retardation)? *Tick all that apply, and indicate the percentage of child/adolescent mental health funding covered by each source, if this information is available:*

FUNDING SOURCE	AVAILABLE (Yes/No)	% on the total of child/adolescent mental health funding
Consumer/ Patient/ Family		
Private insurance		
Tax-based Government Funding		
Social insurance (LAMAL)		
International Grants		
Non-Governmental Organization		
Other:		

2.2. Are there other sources of funding for child and adolescent mental health services?

a. Yes *(Please list here below the top three other sources and percentages)*:

b. No

1. ____ - _______%
2. ____ - _______%
3. ____ - _______%

2.3. What subsidized or free government ancillary benefits are provided to a family who has a child or adolescent with a disabling mental disease? *(Please indicate each condition which is available)*

a. No benefits are provided
b. Disability Pension (_________ /month)
c. Specialized Education Programmes
d. Respite/Practical Help for Caregiver
e. Medical (including Psychiatric) Care
f. Institutional Care
g. Parental Training or Education
h. Other *(please specify)*: ______________________________

---

3. CHILD AND ADOLESCENT MENTAL HEALTH SERVICES (CAHMS*)

*the term "CAMHS" refers to specialist, community-based, multidisciplinary, mental health services delivering medical and psycho-social interventions for children and adolescents with mental health problems and disorders and/or neuropsychiatric/developmental disorders

3.1. How many public CAHMS do you have in your canton? *We mean any organized CAMHS providing care to a specified catchment area with one Director/Consultant.* _____________

3.2. Is there a juvenile justice system for delinquent children and adolescents?

a. Yes
b. No

3.2.1 If yes, does this system have access to a specialist/dedicated/forensic child and adolescent mental health service?

a. Yes
b. No

3.3 Are there specialized educational services available for children and adolescents with:

(1. No areas, 2. Few areas, 3. Many areas, 4. Most areas, 5. All areas)

3.3.1 Behavioral problems	1	2	3	4	5
3.3.2 Learning disabilities	1	2	3	4	5
3.3.3 Speech and language delay	1	2	3	4	5
3.3.4 Deaf/blind children	1	2	3	4	5
3.3.5 Mental retardation	1	2	3	4	5
3.3.6 Children with physical and mental disabilities	1	2	3	4	5
3.3.7 Other (please specify):___________	1	2	3	4	5

3.4 Is there a system of providing community-based outpatient care for children and adolescents with mental disorders or severe behavioral problems?

(please check all that apply choosing among: 1. No services/absent – 2. Insufficient services – 3. Sufficient services with medium/low quality – 4. Sufficient services available with good quality– 5. Excellent/comprehensive service available)

3.4.1 Outpatient departments in hospitals	1	2	3	4	5
3.4.2 Private offices of specialists	1	2	3	4	5
3.4.3 Public health/primary care clinics	1	2	3	4	5
3.4.4 Outpatient clinics	1	2	3	4	5
3.4.5 Day patient programs	1	2	3	4	5
3.4.6 Group homes	1	2	3	4	5
3.4.7 Foster care placements	1	2	3	4	5
3.4.8 Respite care placements	1	2	3	4	5
3.4.9 Other *(please specify):*	1	2	3	4	5

3.5 Is there a provision of inpatient mental health care for mentally ill children and adolescents? *Please indicate the total number of inpatient beds in specific child/adolescent inpatient units in the entire country (general hospital/adult psychiatric hospital), if available.*

a. Yes

b. No

3.5.1 Total pediatric beds allocated to children/adolescents: ______________

3.5.2 Total beds allocated to children/adolescents with mental disorders (child/adolescent psychiatric inpatient units): ______________

3.6 Are CAMHS able to offer a language interpreter if needed when a child/adolescent has high needs for diagnostic assessment and/or care and is unable to speak one of the national languages?

(1. No areas, 2. Few areas, 3. Many areas, 4. Most areas, 5. All areas)

3.6.1 diagnostic assessment	1	2	3	4	5
3.6.2. care	1	2	3	4	5

3.7 On average for how many hours per day are CAMHS treating outpatients open in the usual working days (Monday-Friday) ? _______

3.8 Is there a mobile emergency CAMHS team (i.e. outside of hours CAMHS services are available)? *(1. No areas, 2. Few areas, 3. Many areas, 4. Most areas, 5. All areas)* ___________

3.9 Is there a publication or reference providing information about the organization of CAMHS, or about the epidemiology of child/adolescent mental disorders in your country? ________________________________

____

Please give the reference and/or attach a copy of the publication(s)

4. CHILD AND ADOLESCENT MENTAL HEALTH : HUMAN RESSOURCES

4.1. How many child/adolescent psychiatrists (who have a formal specialization in child/adolescent psychiatry) practice child and adolescent psychiatry in your canton? ___________________

4.1.1 Do you have a cantonal child and adolescent psychiatry training program?

a. Yes, please specify: ______________________

b. No

4.1.2 What is the duration of this training program at a cantonal level? _________

4.1.3 Does the program lead to a certificate of specialization?

a. Yes, please specify: ______________________

b. No

4.1.4 Is child and adolescent psychiatry recognized as an official sub-specialty?

a. Yes, please specify: ______________________

b. No

4.1.5 Is there a compulsory Continuing Medical Education program for child/adolescent psychiatrists?

a. Yes, please specify: ______________________

b. No

4.2 How many clinical psychologists specialized in the area of child/adolescent mental health are there in your canton? ___________________

4.2.1 Do you have a cantonal training program for clinical psychologists working with children and adolescents?

a. Yes, please specify: ______________________

b. No

4.2.2 What is the duration of this training program at a cantonal level? ________

4.2.3 Does the program lead to a certificate of specialization?

a. Yes, please specify: ______________________

b. No

4.2.4 Is there a compulsory Continuing Education program for child/adolescent clinical psychologists?

a. Yes, please specify: ______________________

b. No

4.3 Which other professionals work with children and adolescents with mental disorders?
(Please check all that apply)

4.3.1 Psychiatric nurses	Yes	No
4.3.2 Social workers	Yes	No
4.3.3 Speech and language therapists	Yes	No
4.3.4 Other professionals (please specify):	Yes	No

---

5. COLLABORATION WITH OTHER SERVICES

5.1. Is there any protocol or agreement between schools and health services at the national/cantonal level to refer a child with suspected learning disabilities to a CAMHS?

a. Yes
b. No

5.2. Is there any protocol or agreement between schools and health services at the community/canton/regional level to refer a child with suspected learning disabilities to a CAMHS? *(1. No areas, 2. Few areas, 3. Many areas, 4. Most areas, 5. All areas)* ________

5.3. Is there any specific protocol to let schools, social services, other public and private agencies, etc. signal severe cases of abuse or neglect to mental health care providers? *(1. No areas, 2. Few areas, 3. Many areas, 4. Most areas, 5. All areas)* _________

5.4. Are there any established relationships between CAMHS and services for the protection of children and adolescents from abuse and neglect*? (1. No areas, 2. Few areas, 3. Many areas, 4. Most areas, 5. All areas)* _________

5.5. Do official referral procedures at a national/cantonal level from primary care to secondary/tertiary care exist?

a. Yes
b. No
c. Unknown

5.6. At least one service users' association is present in the country/canton

a. Yes
b. No
c. Unknown

5.7. At least one family/carers' association is present in the country/canton

a. Yes
b. No
c. Unknown

5.8. Are service users' associations/organizations involved in the formulation or implementation of mental health policies, plans or legislation at national/cantonal level in the last two years? *(Consider the presence of at least one representative member of a user association/organization during the meetings for the last revision of the mental health policy/plan or legislation -mark only one item)*

a. unknown
b. NA: service users' associations/organizations do not exist or there is no policy/plan/legislation present in the country
c. never or rarely: service users' associations/organizations did not or rarely participated in the meetings for the formulation/implementation of the policy/plan/legislation
d. not routinely: service users' associations/organizations participated but not routinely in the meetings for the formulation/implementation of the policy/plan/legislation
e. frequently service users' associations/organizations participated routinely in the meetings for the formulation/implementation of the policy/plan /legislation

5.9. Are family/carers' associations/organizations involved in formulation or implementation of mental health policies, plans or legislation at national/cantonal level in the last two years ? *(Consider the presence of at least one representative member of a user association/organization during the meetings for the last revision of the mental health policy/plan or legislation -mark only one item)*

a. unknown
b. NA: family/carers' associations/organizations do not exist or there is no policy/plan/legislation present in the country
c. never or rarely: family/carers' associations/organizations did not or rarely participated in the meetings for the formulation/implementation of the policy/plan/legislation
d. not routinely: family/carers' associations/organizations participated but not routinely in the meetings for the formulation/implementation of the policy/plan /legislation

e. frequently family/carers' associations/organizations participated routinely in the meetings for the formulation/implementation of the policy/plan /legislation

6. ACTIVITY DATA

6.1. Does the national/cantonal health care system require any periodic activity report from CAHMS?

a. Yes

b. No

6.2. How many patients have been treated in all CAHMS operating in your canton in the latest year available? *("treated" meaning those patients with at least one contact with service in the last 3 months, new patients included)*
N°: _______________

6.3. In the latest year how many of them were females and how many males?
Females: __________ *Males:* __________

6.4. What is the age distribution in ranges? *(please specify the age ranges available in your country)*

	N° all patients	N° females	N° males
……….. *years*			
……….. *years*			
……….. *years*			
……….. *years*			
……….. *years*			

6.5. What is the distribution according to DSM-V or to the ICD-10 diagnostic categories? *(Please in case of multiple diagnosis consider the main diagnosis; insert 0 if there are no users in a certain category; if possible, please try to adapt diagnostic categories different from DSM-IV to the current DSM-V system; otherwise use ICD-10 categories)*

DSM-V CATEGORIES			
	Total N°	N° males	N° females
**Neurodevelopmental disorders (please specify) - Intellectual disabilities**<br>- Communication disorders<br>- Autism spectrum disorders<br><br>- Attention deficit/hyperactivity disorder - Specific learning disorders<br>- Motor disorders<br>- Other neurodevelopmental disorders			
**Schizophrenia** spectrum and other psychotic disorders			
**Bipolar** and related disorders			
**Depressive** disorders			
**Anxiety** disorders			
**Obsessive compulsive**-related disorders			
**Trauma-Stress** related disorders			
**Dissociative** disorders			
**Somatic symptoms** and related disorders			
Feeding and **Eating** disorders			
**Elimination** disorders			
**Sleep-wake** disorders			
**Sexual** dysfunctions			
**Gender** dysphoria			
Disruptive, impulse control, and **conduct** disorders			
Substance-related and **addictive** disorders			
**Neurocognitive** disorders			
**Personality** disorders			
**Paraphilic** disorders			
**Other** mental disorder			
**Medication-induced** movement disorders and other adverse effects of medication			

ICD-10 CATEGORIES			
	Total N°	N° males	N° females
F00-F09<br>Organic, including symptomatic, mental disorders			
F10-F19<br>Mental and behavioural disorders due to psychoactive substance use			
F20-F29<br>Schizophrenia, schizotypal and delusional disorders			
F30-F39<br>Mood [affective] disorders			
F40-F48<br>Neurotic, stress-related and somatoform disorders			
F50-F59<br>Behavioural syndromes associated with physiological disturbances and physical factors			
F60-F69<br>Disorders of adult personality and behaviour			
F70-F79<br>Mental retardation			
F80-F89<br>Disorders of psychological development (please specify)<br><br>F80 Specific developmental disorders of speech and language F81 Specific developmental disorders of scholastic skills<br>F82 Specific developmental disorder of motor function<br>F83 Mixed specific developmental disorder<br><br>F84 Pervasive developmental disorders<br>F88 Other disorders of psychological development<br>F89 Unspecified disorder of psychological development			
F90-F98<br>Behavioural and emotional disorders with onset usually occurring in childhood and adolescence			
F99<br>Unspecified mental disorder			

6.6. In the latest year available how many new cases (total no.) have been recorded in all CAMHS active at cantonal level? *(do not consider patients who were discharged and then came back to services, even after a long time, include only first-ever contacts, if this information is available)*

N° new accesses: _________

7. DATA COLLECTION AND QUALITY ASSURANCE

7.1. Is there any epidemiological data collection system for child and adolescent mental health disorders? *(i.e., psychiatric case registers, hospital information systems, etc.)*

a. Yes
b. No

7.1.1 If yes please supply reference details of 3-4 articles that provide epidemiological data on child/adolescent mental health in your country *(prevalence and incidence studies, service utilization studies, suicide rate studies, psychotropic drug utilization studies)*

____

____

____

____

7.2. Is there any service data collection system at national/cantonal level for child and adolescent mental health disorders?

a. Yes
b. No

7.2.1 If yes, is there any regular monitoring of treatment outcomes?

c. Yes
d. No

8. CARE FOR SPECIAL POPULATIONS

8.1. Which subgroups of children and adolescents have access to specially designated mental health services, tailored to the subgroup's unique needs? *(Please check all that apply)*

a. None
b. Minority groups
c. Indigenous people
d. Orphans
e. Runaways/homeless
f. Refugees
g. Children affected by natural or man-made disasters
h. "Seriously emotionally disturbed"
i. Other (please specify): ______________________

---

9. MEDICATION OR OTHER TREATMENT MODALITIES

9.1. Which of the following pharmaceutical drug categories are available to the primary health care system for use in children and adolescents? *Please check all that apply; answer the additional questions where applicable.*

	Available Yes/No	Generic name of the 2 most prescribed drugs
Psychostimulants		
Second Generation Antidepressants		
First Generation Antipsychotics		
Second Generation Antipsychotics		
Anxiolytics/sedatives		
Mood stabilizers		

9.2. What other treatment methods are widely used in child and adolescent mental health care? *(Please check all that apply)*

a. Cognitive-Behavioural Therapy
b. Behavioural modification training
c. Family psycho-education
d. Systemic therapy
e. Social skills training
f. Learning assistance/educational supports
g. Parental training/guidance
h. Home support
i. Speech/language training
j. Other psychotherapies (please specify): _______________
k. Other (please specify): _______________

---

10. SOURCES

What source of information did you use to fill in the questionnaire? *Please specify all) ….*

1. :

2. :

3. :

4. :

5. :

6. :

7. :

8. :

9. :
