# Supplementary material for: Mapping Child and Adolescent Mental Health Services and the Interface During Transition to Adult Services in Six Swiss Cantons
Source: Front Psychiatry. 2022 May 9;13:814147. doi: 10.3389/fpsyt.2022.814147 (PMC9125157; doi:10.3389/fpsyt.2022.814147)
Supplement: Supplementary file 3 [file Data_Sheet_3.docx]

**Supplementary material**

**Appendix 1:** Collaboration between services

|  | **Basel-City** | **Geneva HUG** | **Geneva OMP** | **Fribourg** | **Neuchâtel** | **Valais** | **Vaud** |
| --- | --- | --- | --- | --- | --- | --- | --- |
| Collaboration with schools for children and adolescents with learning disabilities | All areas | No area | All areas | No area | All areas | All areas | Few areas |
| Collaboration with schools, social services, other public and private agencies for children and adolescents to signal severe cases of abuse or neglect | All areas | Most areas | All areas | All areas | All areas | All areas | Most areas |
| Collaboration between services to protect children and adolescents from abuse and neglect | All areas | All areas | All areas | Many areas | Most areas | All areas | All areas |

HUG, Hôpitaux Universitaires de Geneve; OMP, Office Medico-Pedagogique.

**Appendix 2:** Sources of health financing and proportion by canton

| **Funding source** | **Basel-City** | **Geneva HUG** | **Geneva OMP** | **Fribourg** | **Neuchâtel** | **Valais** | **Vaud** |
| --- | --- | --- | --- | --- | --- | --- | --- |
| **Consumer, patient, family** | NA | NA | - | 10% | 10% | 10% | 5% |
| **Private insurance** | NA | NA | - | 15% | - | - | - |
| **Taxe based government funding** | NA | NA | 70% | 50% | 60% | - | 35-40% |
| **Mandatory insurance (LAMaL)** | NA | NA | 30% | 25% | 30% | 80% | 55-60% |
| **International grants** | NA | NA | - | - | - | - | <1% |
| **Non-governmental organization** | NA | NA | - | - | - | - | <1% |
| **Cantonal youth service** | NA | NA | - | - | - | 10% | - |

-no funding by the mentioned source; HUG, Hôpitaux Universitaires de Geneve; NA, information not available; OMP, Office Medico-Pedagogique.

**Appendix 3:** Type of psychological interventions used by canton

|  | **Basel-City** | **Geneva HUG** | **Geneva OMP** | **Fribourg** | **Neuchâtel** | **Valais** | **Vaud** | **Total** |
| --- | --- | --- | --- | --- | --- | --- | --- | --- |
| **Systemic therapy** | Yes | Yes | Yes | Yes | Yes | Yes | Yes | 7 |
| **Parental training ; guidance** | Yes | Yes | Yes | Yes | Yes | Yes | Yes | 7 |
| **Psychoanalysis and psychotherapy** |  | Yes | Yes | Yes | Yes | Yes | Yes | 6 |
| **Family psycho-education** | Yes | Yes | Yes |  | Yes | Yes | Yes | 6 |
| **Home support** | Yes | Yes | Yes |  | Yes | Yes | Yes | 6 |
| **Speech and language training** | Yes | Yes | Yes | Yes |  | Yes | Yes | 6 |
| **Cognitive behavioral therapy** | Yes |  | Yes |  | Yes | Yes | Yes | 5 |
| **Learning assistance and educational support** | Yes |  | Yes | Yes |  | Yes | Yes | 5 |
| **Social skills training** | Yes |  |  |  | Yes | Yes | Yes | 4 |
| **Early intensive intervention in ASD** | Yes |  | Yes |  |  |  | Yes | 3 |
| **Behavioral modification training** | Yes |  |  |  |  | Yes |  | 2 |
| **Eye Movement Desensitization and Reprocessing** |  |  |  |  |  | Yes |  | 1 |

HUG, Hôpitaux Universitaires de Geneve ; OMP, Office Médico-Pédagogique.

**Appendix 4:** Type of medication in CAMHS most commonly used by canton

|  | **Basel-City** | **Geneva** | **Fribourg** | **Neuchâtel** | **Valais** | **Vaud** |
| --- | --- | --- | --- | --- | --- | --- |
| **Psychostimulants** | Methylphenidate | Methylphenidate, Atomoxetine | Methylphenidate | - | Methylphenidate | Methylphenidate, Atomoxetine |
| **Second generation antidepressants** | Fluoxetine, Sertraline | Citalopram, Sertraline | Fluoxetine, Sertraline | - | Sertraline, Escitalopram | Fluoxetine, Sertraline |
| **First generation antipsychotics** | Levomepromazine *(rarely)* | Not used | Haloperidol, Levomepromazine | - | Haloperidol | Haloperidol, Levomepromazine |
| **Second generation antipsychotics** | Risperidone, Quetiapine | Aripiprazole | Risperidone, Aripiprazole | - | Risperidone, Aripiprazole | Risperidone, Aripiprazole |
| **Anxiolytics, sedatives** | Lorazepam | Risperidone,  Quetiapine | Lorazepam, Alprazolam | - | Lorazepam, Alprazolam | Lorazepam |
| **Mood stabilizers** | Valproate | Lithium | Valproate, Carbamazepine | - | Quetiapine,  Aripirazole | Valproate |

-medication is used but no specific medication name was provided.

**Appendix 5:** Transition planning and involvement of transition stakeholders

|  | **Basel-City** | **Geneva HUG** | **Geneva OMP** | **Fribourg** | **Neuchâtel** | **Valais** | **Vaud** |
| --- | --- | --- | --- | --- | --- | --- | --- |
| **Documented hand over planning** | Sometimes | Never | Never | Sometimes | Never | Sometimes | Sometimes |
| **Joint meeting with adult services** | Sometimes | Sometimes | Sometimes | **Always** | Sometimes | **Always** | Sometimes |
| **Involvement of parent/carer in care plan and decision making** | Sometimes | Never | **Always** | **Always** | Sometimes | **Always** | Sometimes |
| **Involvement of young person in care plan and decision making** | Sometimes | Never | Sometimes | Sometimes | **Always** | **Always** | **Always** |
| **Preparing young person for ending therapeutic relationship** | Sometimes | Sometimes | **Always** | **Always** | **Always** | **Always** | **Always** |
| **Accountability of one clinician for the transition process** | Sometimes | Never | Never | **Always** | **Always** | **Always** | Sometimes |

HUG, Hôpitaux Universitaires de Geneve ; OMP, Office Médico-Pédagogique.

**Appendix 6:** Availability of additional support for the management of the “transition period” by canton

|  | **Basel-City** | **Geneva HUG** | **Geneva OMP** | **Fribourg** | **Neuchâtel** | **Valais** | **Vaud** |
| --- | --- | --- | --- | --- | --- | --- | --- |
| **A transition team** | No area | No area | No area | No area | No area | No area | No area |
| **Joint working with adult service providers** | Few areas | Few areas | **Many areas** | Few areas | No area | **All areas** | **Many areas** |
| **Joint working between adult and children's service providers** | Few areas | Few ares | No area | **Many areas** | No area | **All areas** | **Many areas** |
| **Shared documentation and record keeping system (CAMHS/AMHS)** | Most areas | **All areas** | No area | Few areas | No area | **All areas** | No area |
| **Involvement of young person in planning** | No area | No area | No area | No area | No area | **All areas** | **Many areas** |
| **Out of clinic support to move into less structured life of adulthood** | Few areas | No area | No area | No area | No area | No area | No area |

HUG, Hôpitaux Universitaires de Geneve ; OMP, Office Médico-Pédagogique.

**Appendix 7:** Additional programs offered to address the needs of young people who are discharged from the child system or service

|  | **Basel-City** | **Geneva HUG** | **Geneva OMP** | **Fribourg** | **Neuchâtel** | **Valais** | **Vaud** | **Total** |
| --- | --- | --- | --- | --- | --- | --- | --- | --- |
| **Supervised or supported housing** |  |  |  |  | Yes | Yes | Yes | 3 |
| **Vocational support** |  |  | Yes |  |  |  | Yes | 2 |
| **Supported education** |  |  | Yes |  |  |  | Yes | 2 |
| **Transition support service** |  |  | Yes |  |  | Yes |  | 2 |
| **Assertive community treatment** |  |  |  |  |  |  | Yes | 1 |
| **Health promotion** |  |  |  |  |  | Yes |  | 1 |
| **Standard wraparound approaches** |  |  |  |  |  | Yes |  | 1 |
| **Independent living preparation** |  |  |  |  |  |  |  | 0 |
| **Approaches tailored to address transition** |  |  |  |  |  |  |  | 0 |
| **Peer leadership, mentoring** |  |  |  |  |  |  |  | 0 |
| **Transition specialist** |  |  |  |  |  |  |  | 0 |

HUG, Hôpitaux Universitaires de Geneve ; OMP, Office Médico-Pédagogique.
